# Characteristics of total laparoscopic hysterectomy among women with or without previous cesarean section: retrospective analysis

**DOI:** 10.1590/1516-3180.2018.0197030718

**Published:** 2018-10-22

**Authors:** Nadiye Koroglu, Berna Aslan Cetin, Gokce Turan, Gonca Yetkin Yıldırım, Aysu Akca, Ali Gedikbasi

**Affiliations:** I MD. Physician, Department of Obstetrics and Gynecology, Health Sciences University Kanuni Sultan Suleyman Training and Research Hospital, Istanbul, Turkey.; II MD. Physician, Department of Obstetrics and Gynecology, Health Sciences University Kanuni Sultan Suleyman Training and Research Hospital, Istanbul, Turkey.; III MD. Physician, Department of Obstetrics and Gynecology, Kırıkhan State Hospital, Hatay, Turkey.; IV MD. Physician, Department of Obstetrics and Gynecology, Health Sciences University Kanuni Sultan Suleyman Training and Research Hospital, Istanbul, Turkey.; V MD. Physician, Department of Obstetrics and Gynecology, Health Sciences University Kanuni Sultan Suleyman Training and Research Hospital, Istanbul, Turkey.; VI MD. Associate Professor, Department of Obstetrics and Gynecology, Health Sciences University Kanuni Sultan Suleyman Training and Research Hospital, Istanbul, Turkey.

**Keywords:** Laparoscopy, Hysterectomy, Cesarean section, Postoperative complications.

## Abstract

**BACKGROUND:**

The number of hysterectomized patients with previous cesarean sections (CSs) has increased due to increasing CS rates. A previous history of CS has been demonstrated to be an important risk factor for major complications in total laparoscopic hysterectomy. The aim here was to evaluate the major complications and safety of TLH in patients with previous CS.

**DESIGN AND SETTING:**

Retrospective analysis in a tertiary-level center.

**METHODS:**

The medical records of 504 total laparoscopic hysterectomy patients operated between May 2013 and May 2017 were reviewed retrospectively. Data on age, parity, surgical indications, duration of operation, length of hospital stay, histopathological diagnosis and major intra and postoperative complications were gathered. The patients were categorized into two groups according to their CS history, namely those with and those without previous CS. Major complications were defined as the presence of lower urinary tract injury (bladder or ureter injury), enterotomy/colostomy, bowel serosal injury or vascular injury.

**RESULTS:**

There was no difference between the groups in terms of parity, duration of operation, hospital stay or pre and postoperative hemoglobin levels. The conversion rates to laparotomy in the previous CS and no CS groups were 2% and 1.7%, respectively. The rates of major complications in the previous CS and no CS groups were 5% and 1.3%, respectively, and these results did not differ significantly (P > 0.05).

**CONCLUSION:**

TLH could be performed safely in the previous CS group, since the complication rate was not different from that of the patients without previous CS.

## INTRODUCTION

Hysterectomy is one of the most commonly performed gynecological operations. It is carried out because of a variety of indications, such as presence of dysfunctional uterine bleeding, myoma uteri, adenomyosis and adnexal mass. Hysterectomy can be performed using abdominal, vaginal, laparoscopic or robotic methods. According to the results from a study performed in the United States, the incidence rates for hysterectomies using abdominal, vaginal and laparoscopic methods are 66%, 22% and 12%, respectively.^1^

There is still no consensus on which of these approaches is the optimum surgical method for hysterectomy. Abdominal hysterectomy is the most frequently performed approach, but current clinical practice mandates that, when appropriate, the surgical method should be vaginal rather than abdominal, since the former is associated with better outcomes and lower complication rates. Moreover, when vaginal hysterectomy is not feasible or not indicated, the surgical method should be laparoscopic, because total laparoscopic hysterectomy (TLH) provides a faster return to normal activity, shorter hospital stays, lower intraoperative bleeding and fewer wound infections, compared with abdominal hysterectomy. However, longer operating times and higher incidence of urinary system damage are seen in laparoscopic hysterectomies.^2^

Because of the gradually increasing rates of cesarean sections (CSs) over the last two decades, the number of hysterectomized patients with previous CS has increased. In a recent review article, previously performed CSs were demonstrated to be an important risk factor for lower urinary tract injuries, and the recommendation that abdominal hysterectomy might be preferable for these patients was emphasized.^3^ TLH may be technically difficult in patients with previous CSs, due to surgical adhesions, and is associated with a higher risk of perioperative complications.^4^

## OBJECTIVES

To identify the characteristics of women undergoing total laparoscopic hysterectomy, and the frequency of peri and postoperative complications, comparing those with or without previous cesarean section.

## METHODS

### Study design, setting and participants

This was a retrospective analysis that included all patients who had undergone TLH in our hospital between May 2013 and May 2017. The medical records of 695 patients who underwent TLH were reviewed. Among these patients, 150 were excluded because of occurrences of tubo-ovarian abscess, endometriosis, pelvic tuberculosis, pelvic organ prolapses, history of previous abdominal surgeries or gynecological malignancies. Another 40 cases were excluded because of insufficient data in the medical records. The study was approved by the local ethics committee (date: July 31, 2017; approval number: 2017-08-26).

The patients were classified into two groups according to whether or not they had previously undergone CS. Based on their history of CSs, the patients’ medical records were compared with regard to age, parity, body mass index, surgical indications, duration of operation, length of hospital stay and major intra and postoperative complications. Major complications were defined as the presence of lower urinary tract injury (bladder or ureter injury), enterotomy/colostomy, bowel serosal injury or vascular injury.

### Statistical analysis

The statistical analyses were performed using the Statistical Package for the Social Sciences, version 20 (SPSS Inc.). The chi-square and Student’s t tests were used for statistical comparisons. P < 0.05 was considered statistically significant.

## RESULTS

This study was completed with 505 patients. Using Yada-Hashimato’s study as a guide, a power analysis was performed and a total sample size of 500 was estimated as required to obtain a power of 80% for difference between two independent groups (0.4 effect size and 0.05 α error).

All the patients were hospitalized one day before the operation and underwent bowel preparation with liquid enema, overnight before the surgery. Fluid intake was withheld for at least eight hours before the surgery. All the patients were catheterized through the urethral route before the surgery was undertaken, and the catheters were removed on the first postoperative day. The duration of the operation was defined as the time that elapsed from the induction of anesthesia to its termination.

In all cases that underwent TLH, the standard surgical technique was used. In most of the patients, a primary trocar with a caliber of 10 mm was directly inserted through the umbilicus or supraumbilical region, according to the size of the uterus. In situations of failure of access with the primary trocar, an open technique was used. In addition, a total of three accessory trocars with a caliber of 5 mm were inserted suprapubically through the midline and at points on the left and right sides of the midline.

The round ligaments were cauterized and cut bilaterally. The infundibulopelvic ligament was cauterized, and it was then cut in cases requiring oophorectomy. In contrast, in patients whose ovaries would be spared, the utero-ovarian ligaments were cauterized and cut. Bilateral salpingectomy was performed. The anterior and posterior peritoneum was opened. The bladder was dissected, beginning from the left lateral aspect, and was pushed downward.

After these steps, the uterine arteries were skeletonized and cauterized bilaterally. The vaginal manipulator was retracted to identify the cervicovaginal junction. Colpotomy was performed on the rim of the Clermont-Ferrand uterine manipulator, using a monopolar cautery, and the specimen was removed by means of the vaginal route. Finally, the vaginal vault was closed laparoscopically using a barbed no. 2 suture in all cases.

The TLH procedure was performed by eight surgeons who had laparoscopic training certificates or who had performed an average of 30 cases with an experienced surgeon.

It was found that the cases with a history of CS were significantly younger than the cases without previous CS (P = 0.01). Apart from the patients’ ages, the clinical characteristics of the groups were comparable (Table 1). Body mass index, parity, surgical indications, duration of operation and duration of hospitalization were similar between the groups. 67% of the patients with previous CS had undergone one CS, 25% had undergone two CSs and 8% had undergone three CSs. Uterine myoma was the most frequent indication for surgery in both groups; other indications and their frequencies are shown in Table 2. No difference between the two groups was detected in terms of major complications (Table 2). In the multivariate logistic regression analysis, the effect of age on complications was not statistically significant (P = 0.36; RR: 1.03; 95% CI 0.96-1.11).

**Table 1.** Clinical characteristics of patients with no cesarean section (CS) and previous CS

	No CS (n = 446)	Previous CS (n = 59)	P-value
Age (years)	49 ± 6.3	46.8 ± 5.5	0.015
Gravida (n)	4.3 ± 2.5	4.2 ± 2.6	0.753
Parity (n)	3.5 ± 2.1	3.4 ± 2.2	0.825
Body mass index (kg/m^2^)	31.7 ± 5.2	32.7 ± 5.7	0.220
Preoperative hemoglobin (g/dl)	11.5 ± 1.6	11.7 ± 1.5	0.331
Postoperative hemoglobin (g/dl)	10.5 ± 1.5	9.9 ± 1.2	0.785

Data are expressed as mean (± standard deviation).

**Table 2.** Surgical outcomes relating to TLH among patients with no cesarean section (CS) and previous CS

	No CS (n = 446)	Previous CS (n = 59)	P-value
Type of surgery (n, %)			
TLH	225 (50.4)	33 (55.9)	0.428
TLH + BSO	221 (49.6)	26 (44.1)	
Indications (n, %)			0.662
Myoma uteri Abnormal bleeding Endometrial hyperplasia Others	270 (60.5) 84 (18.8) 52 (11.7) 40 (9)	34 (57.6) 14 (23.7) 8 (13.6) 3 (5.1)	0.668 0.372 0.672 0.375
Duration of operation (min)	184.8 ± 52.9	183.7 ± 59.7	0.880
Hospital stay (day)	2.7 ± 1.9	2.7 ± 1.2	0.969
Major complications (n, %) Bladder injury Ureter injury Bowel injury Vessel injury	6 (1.3) 1 (0.2) 0 3 (0.6) 2 (0.5)	3 (5.0) 1 (1.7) 1 (1.7) 1 (1.7) 0	0.218 0.220 0.117 0.393 0.606
Conversion to laparotomy	9 (2.0)	1 (1.7)	0.669

Data are expressed as mean (± standard deviation) or proportions; TLH = total laparoscopic hysterectomy; BSO = bilateral salpingo-oophorectomy.

During TLH, the conversion rates to laparotomy in the previous CS and no CS groups were 2% and 1.7%, respectively. The rates of major complications in the previous CS and no CS groups were 0.16% (n = 6) and 1.21% (n = 3), respectively, and these rates did not differ significantly between the groups (P > 0.05).

In the no CS group, one case of bladder injury, three of bowel injury and two of major vessel injury occurred. In two patients, small retroperitoneal hematomas were observed. Since these were of limited extent, expectant management was implemented. A 3-cm bladder injury defect was repaired laparoscopically. One case of ileum injury, probably due to direct trocar insertion, was detected on the third postoperative day and was treated by means of ileostomy. One case of bowel injury in the rectosigmoid area was detected on the third postoperative day and primary repair was performed. One case of bowel injury in the serosa of the rectosigmoid area was detected intraoperatively and was sutured. In the previous CS group, one case of bladder injury, one of ureter injury and one of bowel injury occurred. One case of bowel injury on the serosal surface of the rectosigmoid colon that was detected intraoperatively was repaired by means of laparoscopic suturing. In addition, a 2-cm bladder injury defect was sutured laparoscopically. One case of ureteral injury was treated by means of double J stent insertion. No vaginal cuff dehiscence occurred in either group.

## DISCUSSION

Many surgeons consider that previous experience of CS increases the incidence of complications relating to hysterectomy. Since TLH allows improved exploration and an opportunity for more delicate dissection, we evaluated the reliability and safety of TLH in patients who had previously undergone CSs. We did not find any significant difference in terms of major complications between the CS and no CS groups. The results from this study demonstrated that TLH is not a risk factor regarding major complications or lower urinary system injuries in cases with a history of CS. In cases with a history of CS, TLH may be performed safely and the complications that may occur, especially those relating to lower urinary system injuries that are managed intraoperatively, are not associated with long-term morbidities.

Moreover, patients in the previous CS group were significantly younger than the cases without CS. This finding is consistent with the results from previous studies.^5^^,^^6^ However, this age difference did not have any impact on complication rates, after adjustment for age in the statistical analysis.

According to the results from an analysis on surgical data conducted in the United States in 2010, the incidence rates for abdominal, laparoscopic and robot-assisted hysterectomy were reported as 65%, 16% and 17%, respectively.^7^ In suitable cases, the minimally invasive vaginal or laparoscopic approach should be preferred, considering the advantages of these approaches over abdominal hysterectomy.^8^ The choice of the route for hysterectomy is affected by many conditions, such as the size and configuration of the uterus and vagina, ease of accessibility (e.g. in situations of descensus or pelvic adhesions), presence of an extrauterine disease, need for additional surgery, surgeon’s experience and clinic’s technical facilities.^9^^,^^10^

No difference between the two groups in this study was observed in terms of major complications (CS group, 5.1%; no CS group, 1.3%). The prevalence rates for bowel injury were 0.6% and 1.7% of the cases with and without previous CS, respectively. In two cases, an intraoperatively detected serosal defect was treated by means of primary suturing, via the laparoscopic approach. In one case, a serosal defect was closed as a primary procedure on postoperative day 3. One case of an ileal defect that was thought to be related to the direct trocar access was detected on postoperative day 3 and repaired with ileostomy.

Most of the major vessel and bowel injuries occurred during intra-abdominal access using a Veress needle or primary trocars.^6^^,^^7^ Although the risk of bowel injury is less than 0.5%, it needs to be borne in mind that if the small bowel is damaged and this is not observed early enough, this condition can increase mortality rates.^9^ The conversion rates to laparotomy in the previous CS and no CS groups were 2% and 1.7%, respectively. The factors that affected conversion to laparotomy included a large uterus that hindered direct vision, laterally located myomas complicating the access to the uterine vessels and previous abdominopelvic surgery.^11^

It is known that previous pelvic surgery may impair the normal anatomy through effacement of the surgical planes. In CSs, adhesions that form between the bladder and uterus complicate dissection during mobilization of the bladder away from the uterus. In cases with previous CS, surgeons often refrain from performing vaginal and laparoscopic hysterectomies for fear of increasing the complication rates.^12^^,^^13^ In a recent cohort study, it was revealed that patients with a history of CS carried a greater risk of hysterectomies over the long term. Moreover, compared with patients who have given birth via vaginal delivery, those with a history of CS more frequently require reoperation after hysterectomy, since they have higher rates of peri and postoperative complications.^14^

A history of CS is the most frequently encountered risk factor for bladder injuries.^15^ The prevalence of bladder injuries has been reported to range between 0.7% and 1.5% among patients who have undergone TLH.^6^^,^^16^ In our study, bladder injury was only seen in one patient (1.7%), who had a previous history of CS, and no statistically significant difference was detected in comparison with those without previous CS. This case of bladder injury was detected intraoperatively and repaired laparoscopically. In cases with TLH, the prevalence of ureteral injuries ranges between 0.04% and 0.70%, representing one-third of the prevalence rate of bladder injuries.^17,18^

Ureteral injuries may be related to the use of electrosurgery for tissue dissection and hemostasis during laparoscopic interventions.^14^ In our study, ureteral injury was detected in one patient in the CS group, while it was not observed in the no CS group. In this case of ureteral injury, ureteral stricture developed secondarily to thermal injury, which was treated through implantation of a double J stent that was left in situ for three months, and no additional surgery was required.

This study had some important limitations. These included its retrospective design and inadequate number of cases in the CS group for evaluation of the rarely seen complications. Since TLHs were performed by more than one surgeon, surgical performance bias might be another limitation of the study. A further limitation was that we did not analyze intraoperative complications according to the level of surgeon training or the surgical case volume performed by the surgeons. In addition, yet another limitation was that we did not evaluate the effect of the number of CSs on the complication rates.

## CONCLUSION

TLH can be performed safely in cases with a history of CSs, since major complication rates were not different from that of the patients without previous CS. Moreover, major complication rates, duration of operation, blood loss, postoperative hospital stays and conversion rate to laparotomy did not differ significantly between patients with and without CS.
